# Gold open access: the best of both worlds

**DOI:** 10.1007/s12471-017-1064-2

**Published:** 2017-12-01

**Authors:** M. A. G. van der Heyden, T. A. B. van Veen

**Affiliations:** Department of Medical Physiology, DH&L, University Medical Center Utrecht, Utrecht, The Netherlands

**Keywords:** Open access, Predatory journal, Impact factor, Article processing charge, Peer review

## Abstract

Gold open access provides free distribution of trustworthy scientific knowledge for everyone. As publication modus, it has to withstand the bad reputation of predatory journals and overcome the preconceptions of those who believe that open access is synonymous with poor quality articles and high costs. Gold open access has a bright future and will serve the scientific community, clinicians without academic affiliations and the general public.

## Open Access publishing and quality control

It is beyond doubt that global scientific progress depends on an open communication of research achievements. Open Access (OA) publishing offers the best guarantee for free access to scientific knowledge for everyone, including peers. Trustworthy OA publishers take huge efforts to assure scientific quality, in contrast to the so-called predatory journals whose mere interest it is to receive the publishing fee, no matter for which content. In general, two flavours of OA exist: green and gold OA. In short, green OA means ‘self-archiving’ of manuscripts, peer-annotated manuscripts, pre-submissions, or peer-reviewed and published works in a freely accessible database. The author should take care not to violate any copyright issues of their published work and avoid double publication. The reader has to realise that not all green OA papers have gone through the traditional peer review cycle of quality control. In contrast, gold OA, in principle, runs via the traditional journal publication cycle of peer review in which the accepted paper will be made freely available but at the expense of a so-called article processing charge (APC). Medical journals, including cardiovascular journals, can be full OA (*e. g. PLOS Medicine, Frontiers in Cardiac Electrophysiology *or *Netherlands Heart Journal*) or hybrid OA that allows authors, following acceptance of their work, to choose for immediate OA or subscription-based access (*e. g. Journal of the American College of Cardiology* or *Cardiovascular Research*). Alternatively, journals may apply delayed OA in which subscription-based journals provide content as OA following an embargo period (*e. g. New England Journal of Medicine*).

## Article processing charge, predatory journals and big deals

Besides the benefits of independent quality assessment and free distribution of knowledge, gold OA still has to deal with some drawbacks and prejudices. Since the business model shifted from subscription to author-based revenues, respected OA journals have to withstand the shadows that predatory journals cast over them [1]. Increasingly, in amount and in frequency, scholars receive e‑mails from malicious journals offering them great chances of rapid publication of their collected research data daily. Because the main, or maybe even sole, purpose of these journals is collecting the APC, peer review, if any at all, is unacceptably superficial and editorial processing is minimal. The Web provides black lists and, in our opinion more valuable, white lists to direct readers and authors to trustworthy OA journals [2]. Currently, new classes of predators in the medical field emerge using methods that go at least one step further as they try to lure scientists with the temptation of obtaining research funding, exampled by journals run by the Pan European Networks company. On their website, this company states that it is ‘devoted to providing the most relevant and up-to-date information for the use of not only the European Commission, but all government agencies and departments across the continent of Europe’. These commercially driven companies will publish your paper, but often at a high cost, and your scientific work will not receive the attention and impact it may deserve. It pays to consider better ways of spending our valuable research money.

In contrast to hybrid and delayed OA journals, full OA publishers may be inclined to lower their quality standards in order to produce more content with the APC in mind. However, from personal experience as a reviewer and editor for full OA journals and as shown by recent studies [3, 4], peer review stringency and subsequent content quality appears not an issue when comparing full OA (*e. g. Frontiers*) [5] and traditional subscription-based peer review journals. Interestingly, OA most likely will increase the number of downloads and the size of readership of a paper, but it does not necessarily mean that the paper will receive more citations. The reason being that those who download and/or read it are not by definition publishing scientists themselves. Subsequently, studies demonstrate that OA has limited or no effect on impact factors [4, 6, 7].

When choosing a journal to submit your work, the APC can be a hurdle. Especially for the very productive research groups, publication costs may affect the research budget to some extent. Although all OA journals have an APC, not all are charging the authors, such as *Netherlands Heart Journal* or *Journal of Biomedical Sciences.* These journals obtain their revenues from external sponsors and the Taiwan Ministry of Science and Technology, respectively. Furthermore, negotiations between scientific publishers and national library consortia resulted in the instalment of so-called ‘big deals’ in a number of countries. These big deals enable authors with a university affiliation to publish their paper in OA free of charge. In the Netherlands, this currently results in free OA publishing in more than 3,500 journals from twelve large publishers (*e. g*. *Elsevier, Springer, Taylor & Francis* and *Wiley*) [8]. Unfortunately, in actual practice it is often up to the authors to inform the publisher of their free OA rights for their accepted manuscript. This calls for improvement.

## Concluding remarks

Gold OA offers freely available, trustworthy scientific knowledge to all. With just a few clicks, individuals active within the respective fields of medicine, including those without any academic affiliation, can easily access important new clinical insights. The general public accessing the internet to find answers to their medical questions will encounter a solid piece of information on which they can rely.
